# A breath of life, and heartbeats for life: the science and soul of neonatal resuscitation^⁎⁎^

**DOI:** 10.1016/j.jped.2025.05.005

**Published:** 2025-06-07

**Authors:** Waldemar A. Carlo, Amy Mackay, Vivek V. Shukla

**Affiliations:** Department of Pediatrics, University of Alabama, Birmingham, AL, United States

Birth asphyxia, defined by the World Health Organization as the failure to initiate and sustain breathing at birth, is the second cause of neonatal mortality and leads to about 25% of neonatal deaths or about 600,000 deaths per year.^1^ While about 50% of all neonatal deaths occur on day 1,^2^ about 75% of deaths due to birth asphyxia occur during the first 24 hours after birth.^3^ Death from prematurity, the most common cause of neonatal mortality overall and during the rest of the first week, accounts for another 900,000 deaths per year.^4^ Poor breathing at birth and inability to sustain breathing contribute to many of the deaths due to prematurity. Thus, effective resuscitation at birth can be one of the most effective interventions to reduce neonatal mortality as it targets 1.5 million neonatal deaths per year due to birth asphyxia and/or prematurity. As neonatal mortality now accounts for almost 50% of under-five mortality,^1^ the provision of timely and effective resuscitation at birth holds promise to reduce the burden of both neonatal and infant mortality in low-resource settings.

Large multicenter clinical trials and meta-analyses of studies in low-resource settings show that neonatal resuscitation and essential newborn care training and practice reduce perinatal mortality. The National Institutes of Child Health and Human Development Global Network for Maternal and Child Health Research simplified versions of the World Health Organization Essential Newborn Care (ENC) program, which included basic resuscitation, and the American Academy of Pediatrics Neonatal Resuscitation Program (NRP). The investigators conducted two large pragmatic implementation and effectiveness trials using an innovative active baseline design appropriate for educational interventions. A large multicenter study conducted in 18 low-risk first-level urban community public sector delivery clinics in Zambia evaluated training midwives sequentially in both simplified programs. In this trial of over 71,000 neonates, essential newborn care training decreased 7-day neonatal mortality by about one-third.^5^ Further training in a simplified NRP resulted in an additional one-third reduction in early neonatal mortality.^5^

Because most neonatal deaths occur in the lowest resource settings, a second trial was conducted in Africa, Southeast Asia, Central America, and South America with most births occurring at home and attended by traditional birth attendants or nurses. This trial, which used both active design and cluster randomized controlled trial components, enrolled over 120,000 births in 96 communities in low-resource rural settings.^6^ Training and implementation of essential newborn care and resuscitation reduced intrapartum stillbirths by about one-third without an increase in neonatal mortality.^6^ The largest trial of neonatal resuscitation (over 277,000 births in 30 facilities in low-resource settings) was a stepped-wedge cluster-randomized implementation study of the Safer Births Bundle of Care program. This trial showed that this program reduced day 1 mortality by over one-third but did not reduce stillbirths.^7^

Meta-analyses of resuscitation training trials in low- and middle-income countries show a reduction in early neonatal mortality, intrapartum-related stillbirths, and perinatal mortality,^8^, ^9^, ^10^, ^11^ but the limited quality of the studies, which in part is intrinsic to educational interventions of resuscitation, may confound the results. For example, a reduction of fresh stillbirths can result in more high-risk survivors who may die during the neonatal period, masking an effect on neonatal mortality. Gaps remain in effective local implementation with delays and interruptions in bag-mask ventilation, a life-saving component of resuscitation.^12^ As outlined in the Utstein formula for resuscitation survival, the three critical components for success in improving neonatal survival include medical science, efficient education, and local implementation.^13^ All three play a role in efforts to address the disparities that remain in neonatal mortality rates.

Effective local implementation is necessary to bridge gaps in quality care. A know-how gap between training and sustained effective bag-mask ventilation performance exists, and knowledge and skills wane over time. Refresher training, local quality improvement, and other evidence-based innovations adapted to the local context to sustain knowledge and skills over time are urgently needed components for local implementation. Quality improvement and implementation research are necessary to bridge the gap between training and clinical practice. Most studies show that neonatal resuscitation training programs improve knowledge, skills performance, and self-efficacy.^14^, ^15^, ^16^ However, there is a loss of knowledge and skills over time^14^ requiring repeat training and practice. Critical skills such as mask ventilation are more difficult to achieve^14^ but can be effective.^15^ Training, including simulation-based training, and frequent refresher training is needed to sustain knowledge and skills, especially in low-resource settings where training is less available.^16^

As in-person neonatal resuscitation training is resource-intensive, and skills wane over time,^14^ online or remote training modalities are needed to increase accessibility and decrease cost. In addition, ongoing locally relevant sustainable practice improvement efforts are needed. In this *Jornal de Pediatria.* Delgado and colleagues^17^ conducted a cluster randomized controlled trial of hybrid distance learning combining online theoretical instruction with face-to-face on-site simulation neonatal resuscitation training compared to traditional face-to-face training. The online component of the training incorporated multimodal digital learning resources, including asynchronous access to structured online theoretical materials, immersive 360° virtual reality clinical simulations, interactive case-based scenarios with real-time feedback mechanisms, and access to the NRP textbook and other e-books. Continuous access to these materials was available. Participants (doctors, nurses, midwives, and nurse technicians) in both groups had access to printed books and inflatable mannequins in their health facilities. Heart rate at 2 minutes after birth was used to assess effectiveness in 2180 births. The results showed that heart rate at 2 minutes was comparable between infants resuscitated by participants in the traditional face-to-face and distance learning methods. Future studies should integrate cost-effectiveness and feasibility analyses together with qualitative assessments of instructor and learner feedback and satisfaction as essential metrics for improving generalizability, adoption, and scalability.

The improvement in heart rate is an objective method to assess the effectiveness of ventilation during neonatal resuscitation. Many lives can be saved by improved effective ventilation, as assessed with heart rate measurements. Most early neonatal deaths are preventable^18^; effective ventilation is essential, and local implementation of effective neonatal resuscitation and essential newborn care is the primary solution to save lives.^8^, ^9^, ^10^, ^11^ The World Health Organization recently made available for free online the revised and exceptional neonatal training programs Essential Newborn Care 1 and Essential Newborn Care 2 which address neonatal resuscitation and other aspects of essential newborn care.^19^ Guidance in setting up local low-dose, high-frequency refresher training and facility-led quality improvement teams and processes is a vital aspect of ENC implementation to sustain skills and improve practices over time.

Effective local implementation of these simple training programs can save about a million lives per year in low-and middle-income countries.^20^ The poet, diplomat, and Nobel Prize laureate Gabriela Mistral wrote, “We are guilty of many errors and many faults, but our worst crime is abandoning the children, neglecting the fountain of life. Many things we need can wait. The child cannot. Now is the time his bones are formed, his mind developed. To him we cannot say tomorrow, his name is today.” There is an urgent need to reduce deaths at birth; the knowledge, training, and tools needed are readily available in high-resource settings, but their inaccessibility accounts for about 10-fold higher mortality of newly born babies in the lowest resource settings where most neonatal deaths occur today. Giving babies the gift of a breath of life at birth can result in heartbeats for life.

## Conflicts of interest

The authors declare no conflicts of interest.
